# Full-length transcriptome and metabolite analysis reveal reticuline epimerase-independent pathways for benzylisoquinoline alkaloids biosynthesis in *Sinomenium acutum*

**DOI:** 10.3389/fpls.2022.1086335

**Published:** 2022-12-20

**Authors:** Yufan Yang, Ying Sun, Zhaoxin Wang, Maojing Yin, Runze Sun, Lu Xue, Xueshuang Huang, Chunhua Wang, Xiaohui Yan

**Affiliations:** ^1^ State Key Laboratory of Component-based Chinese Medicine, Tianjin University of Traditional Chinese Medicine, Tianjin, China; ^2^ Haihe Laboratory of Modern Chinese Medicine, Tianjin, China; ^3^ WuXi AppTec (Tianjin) Co., Ltd., Tianjin, China; ^4^ College of Pharmaceutical Engineering of Traditional Chinese Medicine, Tianjin University of Traditional Chinese Medicine, Tianjin, China; ^5^ School of Medicine, Foshan University, Foshan, Guangdong, China; ^6^ Hunan Provincial Key Laboratory for Synthetic Biology of Traditional Chinese Medicine, Hunan University of Medicine, Huaihua, Hunan, China

**Keywords:** transcriptome, *Sinomenium acutum*, benzylisoquinoline alkaloid, biosynthetic pathway, weighted gene co-expression network analysis

## Abstract

Benzylisoquinoline alkaloids (BIAs) are a large family of plant natural products with important pharmaceutical applications. *Sinomenium acutum* is a medicinal plant from the Menispermaceae family and has been used to treat rheumatoid arthritis for hundreds of years. *Sinomenium acutum* contains more than 50 BIAs, and sinomenine is a representative BIA from this plant. Sinomenine was found to have preventive and curative effects on opioid dependence. Despite the broad applications of *S. acutum*, investigation on the biosynthetic pathways of BIAs from *S. acutum* is limited. In this study, we comprehensively analyzed the transcriptome data and BIAs in the root, stem, leaf, and seed of *S. acutum*. Metabolic analysis showed a noticeable difference in BIA contents in different tissues. Based on the study of the full-length transcriptome, differentially expressed genes, and weighted gene co-expression network, we proposed the biosynthetic pathways for a few BIAs from *S. acutum*, such as sinomenine, magnoflorine, and tetrahydropalmatine, and screened candidate genes involved in these biosynthesis processes. Notably, the reticuline epimerase (REPI/STORR), which converts (*S*)-reticuline to (*R*)-reticuline and plays an essential role in morphine and codeine biosynthesis, was not found in the transcriptome data of *S. acutum*. Our results shed light on the biogenesis of the BIAs in *S. acutum* and may pave the way for the future development of this important medicinal plant.

## 1 Introduction

Benzylisoquinoline alkaloids (BIAs) are a large and diverse group of specialized plant secondary metabolites with important medicinal values. BIAs are widely distributed in the Papaveraceae, Ranunculaceae, Berberidaceae, and Menispermaceae families (Narcross et al., 2016). Pharmacological studies have revealed that BIAs have diverse biological activities, such as anticancer, analgesia, antitumor, and anti-inflammatory (Dang et al., 2012). *Papaver somniferum* is a medicinal plant that produces several pharmaceutically important BIAs, including morphine, codeine, papaverine, and noscapine. It is used as a model organism to investigate the biosynthesis of BIAs (Beaudoin and Facchini, 2014). The recently reported genomes of *Papaver somniferum* (Guo et al., 2018) and *Macleaya cordata* (Liu et al., 2017) provide deep insight into the BIA biosynthetic gene clusters in these medicinal plants and allow the functional characterization of key genes involved in BIA metabolism. Morphine is commonly used as an analgesic drug for pain relief. However, long-term use of morphine can lead to severe health problems, such as drug addiction and abuse (Listos et al., 2019).

Previous studies have shown that various BIAs share similar steps in the early stage of their biosynthesis. The biosynthesis of BIAs begins with the conversion of *L*-tyrosine to two precursors, dopamine and 4-hydroxyphenylacetaldehyde (Sato and Kumagai, 2013). These two compounds are converted by a series of enzymes, such as tyrosine aminotransferase (TyrAT), tyrosine decarboxylase (TYDC), tyrosine/tyramine 3-hydroxylase (3OHase), norcoclaurine synthase (NCS), coclaurine N-methyltransferase (CNMT), and 3′-hydroxyl-*N*-methylcoclaurine 4′-*O*-methyltransferase (4′OMT), to afford (*S*)-reticuline which is the divergent point for the biosynthesis of many BIAs (Hagel and Facchini, 2013). Modification of (*S*)-reticuline by various *O*-methyltransferase (OMTs) and cytochrome P450 oxygenases (CYPs) can lead to the formation of more than 2,500 BIAs. Reticuline epimerase (REPI) is responsible for the conversion of (*S*)-reticuline to (*R*)-reticuline, which is essential for the formation of morphine and codeine (Catania et al., 2022). Apart from simple methylation and oxidation, biosynthesis of some BIAs also undergoes complex reactions, including methylenedioxy bridge formation and phenol coupling. For example, CYP80G2 from *Coptis japonica* can catalyze the intramolecular C–C phenol coupling reaction of (*S*)-reticuline to generate (*S*)-corytuberine. CYP719B1 from *P*. *somniferum* can catalyze the C–C phenol-coupling reaction in morphine biosynthesis (Gesell et al., 2009).

*Sinomenium acutum* is a traditional Chinese herb of the Menispermaceae family. The root and stem of *S. acutum* have long been used to treat rheumatoid arthritis in south China (Liu et al., 2018). Compounds from *S. acutum* have shown anti-inflammatory, analgesic, sedative, and immunosuppressive effects (Liu et al., 2019; Xu et al., 2021a). Sinomenine is the representative compound of *S. acutum*, structurally similar to morphine (Jiang et al., 2020). Sinomenine has preventive and therapeutic effects on opioid dependence without detectable drug addiction (Jin et al., 2008; Bhambhani et al., 2021). The studies on *S. acutum* mainly focused on the chemical constituents and the biological activities of the isolated BIAs (Jiang et al., 2019; Liu et al., 2021), while the biosynthesis of BIAs in *S. acutum* remains untouched, mainly due to the lack of genomic or transcriptomic data.

With the development of high-throughput sequencing technology, transcriptome sequencing has become a powerful tool for studying the regulation of gene expression and the biosynthetic pathways for secondary metabolites in plants (Wang et al., 2019; Zhang et al., 2020a). Combining transcriptomics data with metabolomic analysis is an effective way to identify genes involved in secondary metabolite biosynthesis. This approach has been successfully used in several plant species, such as *C*. *deltoidei* (Zhong et al., 2020), *Stephania tetrandra* (Zhang et al., 2020b), *Polygonatum cyrtonema* Hua (Wang et al., 2019), and *Corydalis yanhusuo* (Xu et al., 2021b).

In this study, we combined the next-generation sequencing (NGS) and single-molecule real-time (SMRT) sequencing techniques to obtain the *de novo* transcriptome assembly of *S. acutum*, aiming to identify the candidate genes involved in the biosynthesis of various BIAs in this plant. The contents of alkaloids in the root, stem, leaf, and seed were determined to help the screening of candidate genes involved in BIA biosynthesis. The transcripts were annotated using various public databases and evaluated by the weighted gene co-expression network analysis (WGCNA) and differentially expressed gene (DEG) analysis to screen candidate genes involved in BIA biosynthesis in *S. acutum*.

## 2 Materials and methods

### 2.1 Plant materials

**Figure 1:**
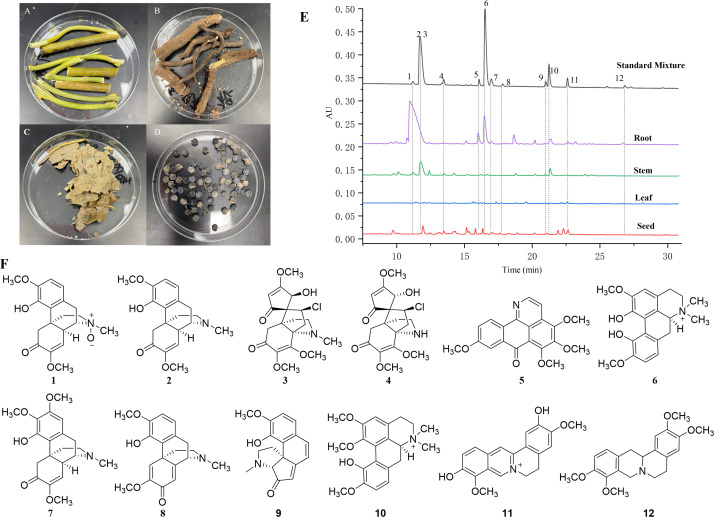
Photos and HPLC profiles of fresh tissues of *S. acutum*. **(A–D)**: the photos of fresh root **(A)**, stem **(B)**, leaf **(C)**, and seed **(D)**. **(E)**: HPLC profiles of the four tissues and the mixed standards of 12 BIAs from *S. acutum*. **(F)**: Chemical structures of 12 selected BIAs, including sinomenine *N*-oxide (1), sinomenine (2), acutumine (3), dauricumidine (4), bianfugenine (5), magnoflorine (6), 8-demethoxyrunanine (7), sinoacutine (8), sinoracutine (9), menisperine (10), stepharanine (11), and tetrahydropalmatine (12).

*Sinomenium acutum* was collected in September 2019 from Huangyan District, Huaihua, Hunan Province, China (27°27’N, 110°40’E) and identified by Dr. Wang Chunhua. Three biological replicates of root (YXH-ZQ-G), stem (YXH-ZQ-J), leaf (YXH-ZQ-Y), and seed (YXH-ZQ-Z) were collected randomly from fresh plants (**Figures 1A–D**). After the collection, the fresh samples were frozen immediately in liquid nitrogen, stored at -80°C, and subjected to sequencing and metabolomic analysis.

### 2.2 Identification and quantification of BIAs

The BIAs in *S. acutum* were identified and quantified using the Waters ACQUITY Ultra-Performance Liquid Chromatography (UPLC) System (Waters, Milford, MA, United States) and the Q Exactive Plus Orbitrap Mass Spectrometer (Thermo Fisher Scientific., Waltham, MA, United States). Dried powder samples (0.25 g) of the root, leaf, stem, and seed of *S. acutum* were accurately weighted and subsequently extracted with 20 mL of methanol solution for 30 min in an ultrasonic bath. Chromatographic separation was carried out on the Waters ACQUITY UPLC system equipped with a BEH C18 column (2.1 × 100 mm, 1.7 µm), and the column temperature was kept at 30°C. The mobile phases were water with 0.1% formic acid (A) and acetonitrile (B) at a flow rate of 0.3 mL/min. The solvent gradients for B were 0–6 min, 5%; 6–20 min, 5–16%; 20–22 min, 16–95%; 22 –25 min, 95%; and 25–26 min, 5%. The Q Exactive MS system used the electrospray ionization source (ESI) in the positive ion mode. The spray voltage and capillary temperature were 3.0 kV and 320 °C, respectively. The flow rates of the atomization gas and heating auxiliary gas were set at 35 arb and eight arb, respectively. The auxiliary gas heating temperature was set at 350°C.

### 2.3 RNA extraction, library preparation, and Illumina Hiseq sequencing

Total RNA from the root, leaf, stem, and seed of *S. acutum* was isolated from the tissue using the TRIzol^®^ Reagent according to the manufacturer’s instructions (Invitrogen, Carlsbad, CA, United States), and genomic DNA was removed using DNase I (Takara, Akasaka, Tokyo, Japan). Then the RNA quality was determined using 2100 Bioanalyser (Agilent, Palo Alto, CA, United States) and quantified using the ND-2000 (NanoDrop Technologies, Wilmington, DE, United States). High-quality RNA samples with OD_260/280_ ≥ 1.8 and the RNA Integrity Number ≥ 6.5 were used to construct the sequencing library and to calculate the Q20, Q30, and GC percentages for each sample. RNA-seq transcriptome libraries were prepared following the TruSeq™ RNA sample preparation Kit from Illumina (San Diego, CA, United States), using 1 μg of total RNA. Shortly, messenger RNA was isolated with polyA selection by oligo(dT) beads and fragmented using a fragmentation buffer. cDNA synthesis, end repair, A-base addition, and ligation of the Illumina-indexed adaptors were performed according to Illumina’s protocol. Libraries were then size-selected for cDNA target fragments of 200–300 bp on 2% Low Range Ultra Agarose followed by PCR amplified using Phusion DNA polymerase (New England Biolabs, Ipswich, MA, United States) for 15 PCR cycles. After quantified by TBS380, the paired-end libraries (150 bp*2) were sequenced using the Illumina NovaSeq 6000 system (Biozeron, Shanghai, China).

### 2.4 *De novo* sequence assembly and gene annotation

The raw paired-end reads were trimmed and quality controlled by Trimmomatic (Bolger et al., 2014). RNA was then assembled *de novo* using Trinity on the clean data from all samples (Grabherr et al., 2011). All assembled transcripts were searched with BlastX against the NCBI protein non-redundant (NR), String, and KEGG databases to identify proteins with the highest sequence similarity to a given transcript to retrieve its functional annotation. A typical *E* value cutoff of less than 1.0 × 10^-5^ was used to define a hit. The Blast2GO program (Conesa et al., 2005) was used to obtain gene ontology (GO) annotations of uniquely assembled transcripts to describe biological processes, molecular functions, and cellular components. Metabolic pathway analysis was performed using the Kyoto Encyclopedia of Genes and Genomes (KEGG).

### 2.5 Analysis of differentially expressed genes

To identify differentially expressed genes (DEGs) between two different samples, the expression level of each transcript was calculated according to the reads per kilobase of exon per million mapped reads (RPKM) method. The RSEM software was used to quantify gene and isoform abundances (Li and Dewey, 2011). The *R* statistical package software was utilized for differential gene expression analysis. In addition, functional-enrichment analysis, including GO and KEGG, was performed to identify which DEGs were significantly enriched in GO terms and metabolic pathways at Bonferroni-corrected *P*-value ≤ 0.05 compared with the whole-transcriptome background. GO functional enrichment and KEGG pathway analysis were carried out by Goatools (Klopfenstein et al., 2018) and KOBAS 2.1.1 (Bu et al., 2021).

### 2.6 Phylogenetic analysis

Phylogenetic analysis of the CYPs and OMTs (4′-OMT, 6-OMT, CoOMT, and SOMT1) involved in BIA biosynthesis in *S. acutum* and other plants were performed based on the deduced amino acid sequences. The alignment was implemented using the MUSCLE algorithm with default parameters. MEGA6 was used to construct the neighbor-joining trees using a bootstrap method with a Poisson model and pairwise deletion (1000 replications).

### 2.7 Weighted gene co-expression network analysis

Weighted gene co-expression network analysis (WGCNA) was performed to identify the key genes involved in BIA biosynthesis in *S. acutum*. WGCNA constructs networks using the absolute value of Pearson’s correlation coefficient as the measure of gene co-expression, which is raised to a power to create the adjacency matrix. The topological overlap distance calculated from the adjacency matrix is clustered with the average linkage hierarchical clustering. Our modules were defined using the cutreeDynamic function with a minimum module size of 30 genes. A module eigengene distance threshold of 0.25 was used to merge highly similar modules using the mergeCloseModules function. We empirically set the cutoff of the weight value as 0.10 to determine biologically significant edges for each module. Correlation analysis between module eigengenes and measured agronomic traits was applied to explore the biological significance of each module.

### 2.8 Quantitative real-time PCR analysis

cDNA was synthesized from 1 µg of total RNA using the 5x PrimerScript RT reagent Kit (Takara Bio Inc). In brief, 10 µL of genomic DNA-removed template, 4 µL of 5x PrimerScript buffer, 1 µL of RT enzyme mix, 1 µL of RT primer, and 4 µL of nuclease-free water were added to result in a 20 µL reverse transcription solution. The mixture was incubated for 30 min at 37°C, followed by inactivated for 5 s at 85°C to afford the cDNA for quantitative real-time PCR (qRT-PCR) experiments. The qRT-PCR was performed in triplicate on 8-strip PCR tubes (Axygen) on a SLAN-96P Real Time PCR System (StrongMed Corporation) using the SYBR Green Mix (Life Technologies). Each PCR solution consisted of 10 µL of SYBR Green Mix, 2 µL of primer mix (10 mM), 5 µL of template cDNA, and 3 µL of nuclease-free water, to obtain a 20 µL system for each PCR reaction. The PCR conditions were: 15 min at 95°C, followed by 40 cycles of 30 s at 95°C, 20 s at 55–65°C, 20 s at 72°C. **Table S2** provides the detailed information about the primer sequences and temperatures. The β-actin gene was used as an internal control for the qRT-PCR analysis.

## 3 Results

### 3.1 Quantification analysis of BIAs in different parts of *S. acutum*

It has been reported that *S. acutum* contains more than one hundred alkaloids, including morphinans, aporphines, protoberberines, benzylisoquinolines, and other compounds. We collected fresh samples of the root, stem, leaf, and seed of *S. acutum* (**Figures 1A–D**). In order to correlate the distribution of alkaloids with the expression of different biosynthetic genes, we first determined the concentration of representative alkaloids in various tissues of *S. acutum* by Q Exactive high-resolution MS and UPLC analysis, respectively (**Figure 1E**). The contents of sinomenine *N*-oxide (**1**, 0.96 ± 0.08 mg/g, DW), sinomenine (2, 22.11 ± 0.09 mg/g, DW), acutumine (3, 0.94 ± 0.02 mg/g, DW), magnoflorine (6, 5.04 ± 0.08 mg/g, DW), 8-demethoxyrunanine (7, 1.26 ± 0.53 mg/g, DW), and tetrahydropalmatine (12, 4.00 ± 0.53 mg/g, DW) were much higher in the root than the other three parts (**Figure 1F** and **Table 1**). Besides the root, sinomenine is also accumulated in the stem. The stem contains most menisperine (10), whereas the concentrations of dauricumidine (4) and sinoracutine (9) are the highest in the seed. Compared to the root, stem, and seed, the leaf contains the least BIAs. We could only detect a trace amount of acutumine (3, 0.07 ± 0.01 mg/g, DW), sinoracutine (9, 0.08 ± 0.01 mg/g, DW), and stepharanine (11, 0.06 ± 0.01 mg/g, DW) in the leaves of *S. acutum*. These results confirmed that the biosynthesis and accumulation of different alkaloids vary significantly in different tissues of *S. acutum*, thus enabling the prediction of genes involved in alkaloid biosynthesis in this plant.

**Table 1:** The BIA contents in different tissues of *S. acutum*.

Alkaloids	Contents (mg/g)			
	Root	Stem	leave	Seed
sinomenine *N*-oxide (**1**)	0.96 ± 0.08	ND	ND	ND
sinomenine (**2**)	22.11 ± 0.25	2.50 ± 0.03	ND	0.09 ± 0.01
acutumine (**3**)	0.94 ± 0.02	0.63 ± 0.05	0.07 ± 0.01	0.64 ± 0.06
dauricumidine (**4**)	0.25 ± 0.01	0.21 ± 0.01	ND	0.48 ± 0.03
bianfugenine (**5**)	0.04 ± 0.01	ND	ND	ND
magnoflorine (**6**)	5.04 ± 0.08	ND	ND	ND
8-demethoxyrunanine (**7**)	1.26 ± 0.11	ND	ND	ND
sinoacutine (**8**)	0.14 ± 0.01	0.03 ± 0.01	ND	ND
sinoracutine (**9**)	0.11 ± 0.01	0.16 ± 0.01	0.08 ± 0.01	0.29 ± 0.01
menisperine (**10**)	0.90 ± 0.01	1.37 ± 0.03	ND	ND
stepharanine (**11**)	0.10 ± 0.01	ND	0.06 ± 0.01	0.19 ± 0.01
tetrahydropalmatine (**12**)	4.00 ± 0.53	0.26 ± 0.01	ND	ND

ND, not detected.

### 3.2 Transcriptome analysis of *S. acutum* samples

**Figure 2:**
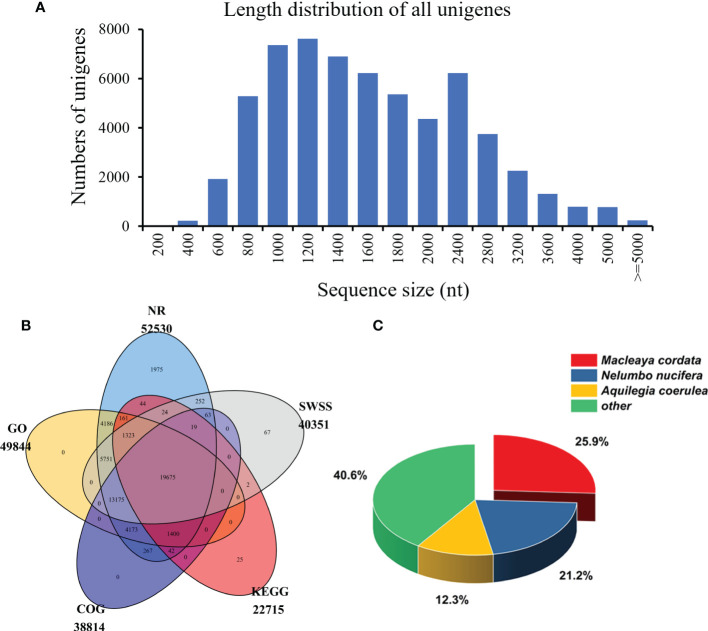
The length distribution and functional annotation of the unigenes in *S. acutum*. **(A)** Length distribution of the 60,675 unigenes. **(B)** Annotated unigenes from different public databases. **(C)** NR homologous species distribution analysis.

After the quantitative analysis, we obtained the transcriptome data of *S. acutum* by performing the single-molecule real-time (SMRT) sequencing on the PacBio Sequel platform and the second-generation sequencing (SGS) on the Illumina platform. Twelve high-quality RNA samples from the root, stem, leaf, and seed of *S. acutum* were sequenced using the Illumina Hiseq system. The SGS sequencing produced average raw reads ranging from 42,406,048 to 46,677,278 and clean reads from 39,799,353 and 43,421,740, respectively. After filtering out the adapters and low-quality reads, the PacBio Sequel platform generated 25,660,172 subreads (34.72 Gb subreads base). The Q20 and Q30 statistics of the clean reads in all samples were higher than 98.2% and 94.2%, respectively, indicating that the sequencing data were of high quality and met the analysis requirements. The average length of the transcripts was 1,353 bp, and the N50 length of all subreads was 1,514 bp. A total of 458,090 circular consensus sequences (CCSs) were detected, with an average read length of 1,645 bp and an N50 of 1,856 bp. We detected 280,999 full-length nonchimeric (FLNC) reads with a mean length of 1,620 bp. Correction of data from the PacBio Sequel platform using the Illumina platform generated 110,876 polished consensus sequences, with an average length of 1,453 bp, an N90 of 861 bp, and an N50 of 1,658 bp, respectively. To analyze the functions of the unigenes from the *S. acutum* transcripts, the redundant sequences were removed *via* the CD-Hit program, and the consensus transcripts were finally clustered into 60,675 non-redundant unigenes, with a mean length of 1,614 bp and an N50 length of 1,840 bp (**Table 2**). Most unigenes are distributed between 800 to 2,800 nt, indicating a high-quality transcriptome of *S. acutum* for future studies (**Figure 2A**).

**Table 2:** Summary of the assembly and annotation of *S. acutum* transcriptome.

	Root	Stem	Leaf	Seed
Illumina sequencing				
Total reads	46,105,271	46,677,278	44,909,644	42,406,048
Nucleotides (nt)	6,915,790,700	7,001,591,800	6,736,446,600	6,360,907,200
Clean reads	42,879,224	43,421,740	42,014,966	39,799,353
Clean nucleotides (nt)	6,404,310,071	6,484,998,541	6,275,854,255	5,948,595,887
PacBio sequencing				
Subreads number	25,660,172			
Average subreads length (nt)	1,353			
N50 length (nt)	1,514			
CCS reads number	458,090			
Average CCS length	1,645			
FLNC reads	280,999			
Unigene	60,675			
Total length (nt)	97,935,561			
N50 (nt)	1,840			
Nr	52,530 (86.6%)			
GO	48,944 (82.2%)			
COG	38,814 (63.9%)			
KEGG	22,715 (37.4%)			
SWSS	40,351 (66.5%)			
All annotated unigenes	52,624 (86.7)			

### 3.3 Functional annotation of the *S. acutum* transcriptome data

The unigenes from the *S. acutum* transcriptome were annotated with the commonly used databases, including the NCBI non-redundant (NR), Swiss-Prot, Clusters of Orthologous Groups of proteins (COGs), Kyoto Encyclopedia of Genes and Genomes (KEGG), and GO (Gene Ontology), using BLASTX (*e* value < 10^-5^). Protein function was predicted according to the annotations of the most similar proteins in these databases. Out of the 60,675 unigenes, 19,675 (32.4%) were annotated by all databases, and 52,624 (86.7%) were annotated by at least one of these databases. The unigenes annotated in the NR, GO, COG, KEGG, and SWSS databases were 52,530 (86.6%), 49,844 (82.2%), 38,814 (63.9%), 22,715 (37.4%), and 40,351 (66.5%), respectively (**Table 2** and **Figure 2B**). KEGG analysis revealed that 5,897 unigenes were predicted to participate in the metabolic pathways, and 3,132 unigenes were involved in the biosynthesis of secondary metabolites (**Figure S1**). KEGG metabolic pathway analysis showed that the unigenes from *S. acutum* could be assigned to 322 pathways (**Supplementary Table 1**). Based on the results of the aligned transcripts in the NR database, 25.9%, 21.2%, and 12.3% of the annotated unigenes had high sequence similarities to the unigenes from *Macleaya cordata*, *Nelumbo nucifera*, and *Aquilegia coerulea*, respectively (**Figure 2C**). It has been reported that these plants all contain a series of alkaloids, such as sanguinarine, chelerythrine, nuciferine, magnoflorine, and aporphine alkaloids (Hagel and Facchini, 2013). The 49,844 unigenes annotated with the GO database were divided into three categories: biological process, molecular function, and cellular component. The largest GO groups in the “biological process” ontology were the “cellular process” and “metabolic process,” with 32,556 and 25,989 annotated unigenes, respectively (**Figure S2**).

### 3.4 Analysis of differentially expressed genes among differenttissues of *S. acutum*

**Figure 3:**
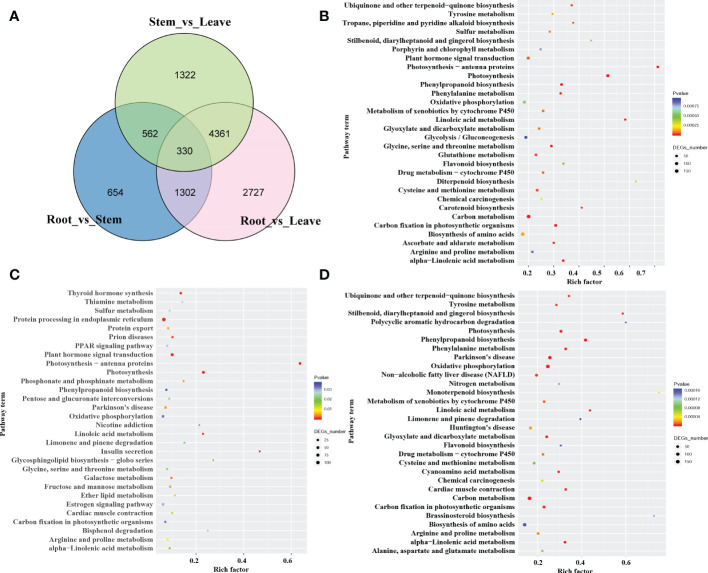
The number and pathway terms of differentially expressed genes among the root, stem, and leaf. **(A)** Venn diagram of DEGs in the three tissues. **(B)**, **(C)**: pathway enrichment of genes predominantly expressed in the root **(B)**, stem **(C)**, and leaf **(D)**.

To investigate the mechanism behind the tissue-specific distribution of various alkaloids, we performed the differentially expressed genes (DEGs) analysis among the root, stem, leaf, and seed in *S. acutum*. The transcript abundance of all the unigenes was evaluated by converting read counts to reads per kilobase of exon model per million mapped reads (RPKM). RNA-seq analysis showed that 35,959, 36,927, 36,086, and 36,975 unigenes in the full-length transcriptome were expressed with RPKM > 1 in the root, stem, leaf, and seed, respectively (**Table S3** and **Figure S3**). A total of 21,594 DEGs were screened under the condition of false discovery rate (FDR) metric with adjusted *p-value* <= 0.05 and | log2(FoldChange) | >= 1. As the quantitative studies have shown that the root of *S. acutum* contains the most alkaloids and the leaf comprises the least alkaloids, we analyzed the DEGs in the root versus the stem and leaf. There are 8,720 DEGs in the root vs. leaf group and 10,026 DEGs in the root vs. seed group. Most of the DEGs are down-regulated in the root. In contrast, only 2,848 DEGs were screened in the root vs. stem group, indicating that the gene expression patterns in the root and stem are much more similar than the other two tissues (**Figure S4**). Of the 8,720 DEGs in the root vs. leaf group, 5,099 genes were down-regulated, and 3,621 genes were up-regulated in the root. Moreover, 330 DEGs were found in the three comparison groups (root vs. stem, root vs. leaf, and stem vs. leaf) (**Figure 3A**). The identified DEGs from different tissues were further analyzed by KEGG enrichment analysis. The significantly enriched pathways are protein processing in the endoplasmic reticulum, carbon metabolism, biosynthesis of amino acids, and plant hormone signal transduction (**Figures 3B–D**). Based on KEGG analysis, 22,715 unigenes were annotated to 6 main categories and 322 biological pathways (**Supplementary Table 1**). In addition, a total of 672 unigenes were assigned to the subcategory “Other secondary metabolite biosynthesis,” of which 247 genes were assigned to “Phenylpropane biosynthesis” (ko00940) and 47 genes to “Isoquinoline alkaloid biosynthesis” (ko00950) (**Table S1**). According to the distribution characteristics of the target alkaloids and the gene expression patterns, the candidate genes involved in alkaloid biosynthesis could be retrieved. These results also align with the previous results that the root and the stem had similar alkaloid contents, and the seed and leaf contain much fewer alkaloids than the root and stem.

### 3.5 Weighted gene co-expression network analysis of the DEGs in *S. acutum*

**Figure 4:**
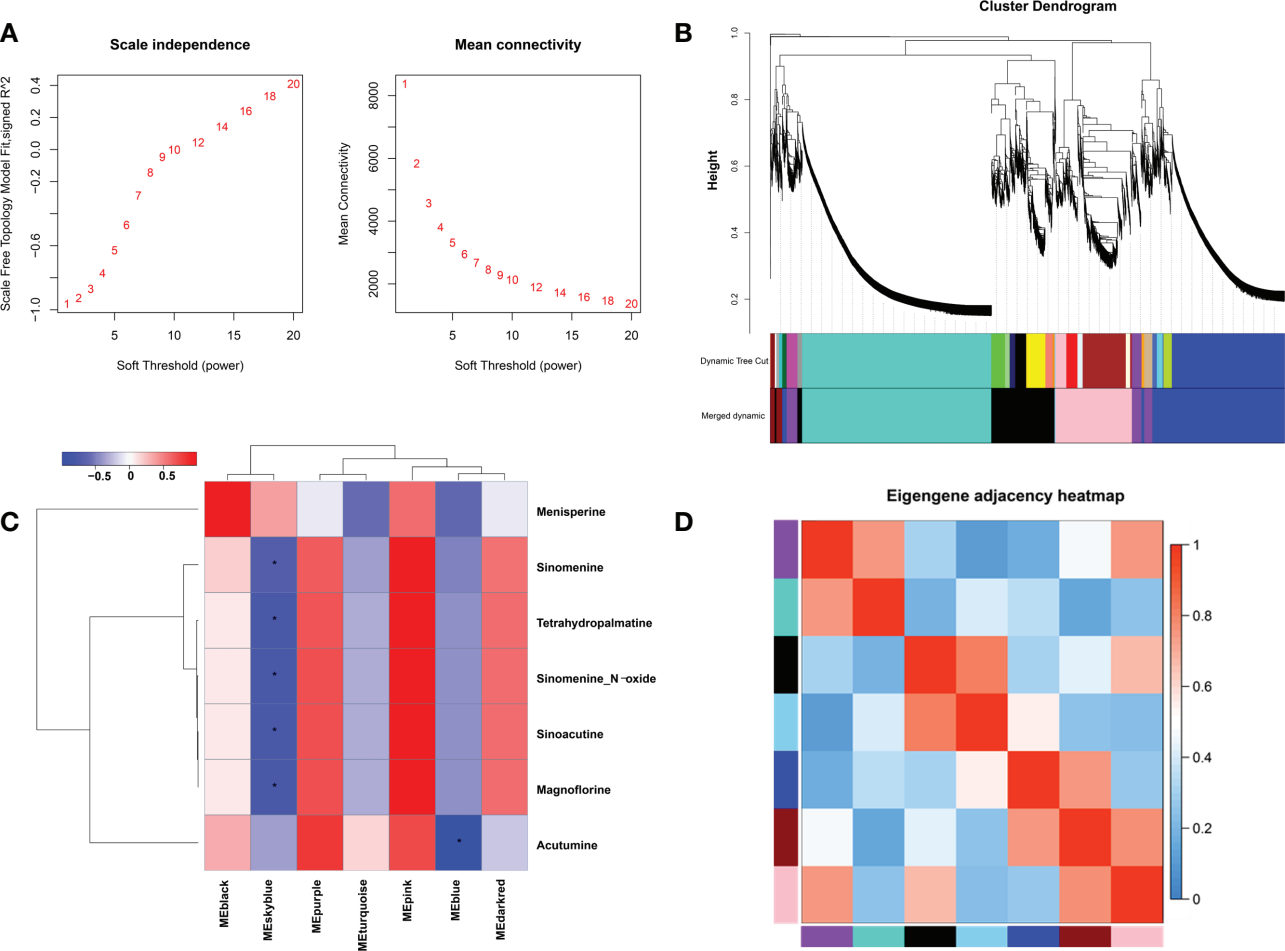
Weighted gene co-expression network analysis (WGCNA) of DEGs identified in the root, stem, leaf, and seed of *S. acutum*. **(A)** Determination of soft-thresholding power in WGCNA. **(B)** Hierarchical cluster tree showing seven modules of co-expressed genes. **(C)** Correlations of the modules and the seven BIAs. **(D)** Heatmap of the correlation between different modules in the weighted gene co-expression network. The symbol * denotes correlation coefficient smaller than -0.5.

In order to explore key genes and co-expression networks that play essential roles in the biosynthesis of alkaloids, we analyzed 15,580 DEGs from 12 samples by weighted gene co-expression network analysis (WGCNA). The optimal soft threshold was set at 12 to construct a scale-free network. The adjacency and tom overlap matrix were established using the function adjacency and tom similarity, respectively [33]. The modules were divided according to the dynamic cutting tree, and the modules with high similarities were merged, leading to the formation of seven modules (**Figures 4A**, **B**). Seven characteristic BIAs of *S. acutum*, including sinomenine, menisperine, tetrahydropalmatine, sinomenine *N*-oxide, sinoacutine, magnoflorine, and acutumine, were used as traits to correlate with the seven gene modules generated by WGCNA analysis. A labeled heatmap function was used to visualize and analyze the relationship between the modules and BIAs. As shown in **Figure 4C**, the pink, purple, and dark red modules were positively correlated with the seven BIAs. The pink, purple, and dark red modules contain 2314, 867, and 307 genes, respectively. The pink, purple, and dark red modules had correlation coefficients of 0.98, 0.63, and 0.53 to sinomenine, and the correlation coefficients of the pink, purple, and dark red modules to magnoflorine were 0.96, 0.67, and 0.56, respectively **(** **Figure 4C**). The eigenvector correlation analysis between each module was performed for all seven modules. It can be seen that the pink and dark red modules and the pink and purple modules have significant correlations (**Figure 4D**). We proposed that the genes in these three modules were more likely to participate in the biosynthesis of BIAs in *S. acutum*.

### 3.6 Identification of candidate genes involved in BIA biosynthesis in *S. acutum*

**Figure 5:**
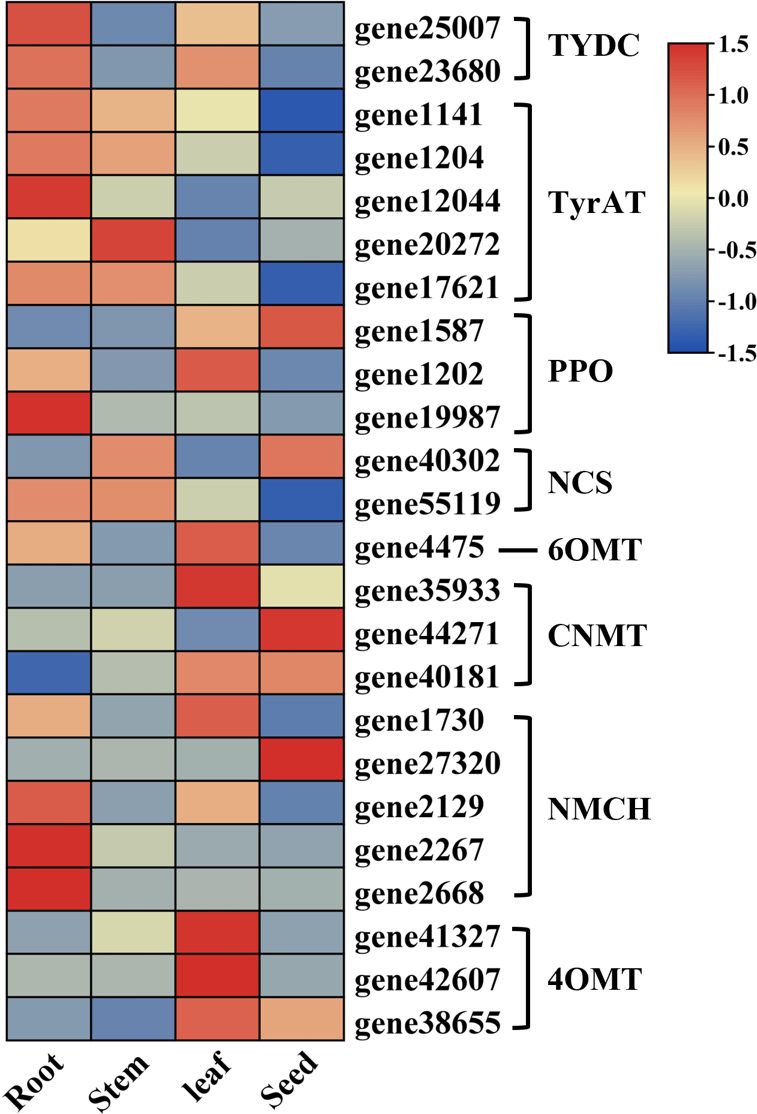
Expression patterns of candidate unigenes in the common biosynthetic pathway of BIAs.

In order to reveal the BIA biosynthetic pathways in *S. acutum*, we divided the pathways into two parts: 1) the common pathway that converts tyramine and 4-hydroxyphenylpyruvate to (*S*)-reticuline and 2) the downstream pathways that drive (*S*)-reticuline to the various BIAs. Based on the results from KEGG pathway analysis and WGCNA screening of the transcriptome data, as well as BLASTP analysis of the unigenes using the benzylisoquinoline biosynthetic enzymes from *P. somniferum* as queries, 24 unigenes encoding enzymes of the common pathway in *S. acutum* were obtained (**Figure 5**). For the classification of cytochrome P450 enzymes, proteins with greater than 40% sequence similarity are grouped into the same family. We also used this criteria to mine the protein homologs for BIA biosynthesis in *S. acutum*. All of the encoded enzymes showed high sequence similarities (similarities > 40%) to the query enzymes from *P. somniferum*. Five candidate genes were found to encode proteins of the TyrAT family and the (*S*)-*N*-methylcoclaurine 3′-hydroxylase (NMCH) family, respectively. In contrast, only one candidate gene (gene4475) was found for the 6OMT.

**Figure 6:**
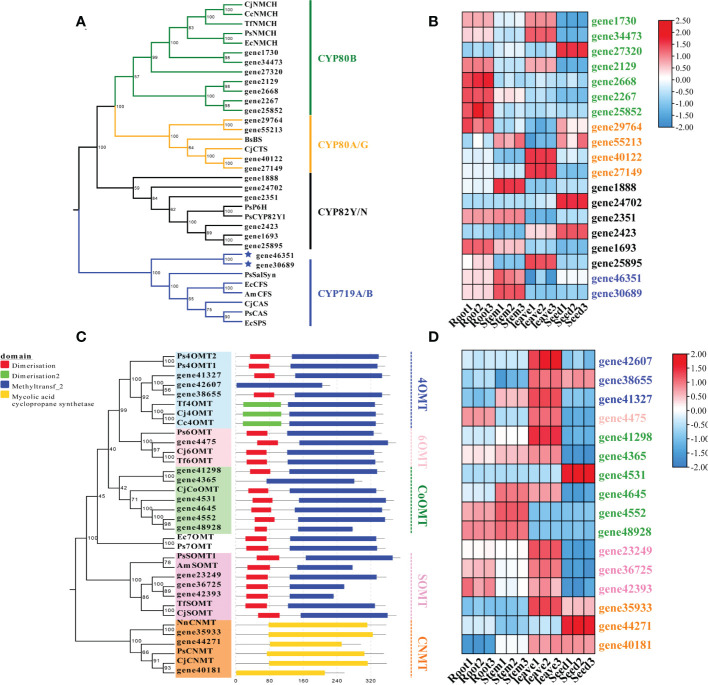
Phylogenetic analysis and expression patterns of CYPs and methyltransferases in *S. acutum*. **(A)** Phylogenetic tree of the candidate CYPs with known CYPs involved in BIA biosynthesis. **(B)** Expression levels of the screened CYP-encoding genes in the root, stem, leaf, and seed of *S. acutum.* **(C)** Phylogenetic tree of the candidate methyltransferases for BIA biosynthesis. **(D)** Expression levels of genes encoding the methyltransferases for BIA biosynthesis in the root, stem, leaf, and seed of *S. acutum.*.

The cytochrome P450 (CYP450) oxygenases and O-methyltransferases were screened for the downstream pathways using Pfam annotation. A total of 262 CYP450-encoding unigenes were found in the transcriptome data of *S. acutum*. Three CYP families, i.e., CYP80, CYP82, and CYP719, have been reported to participate in BIA biosynthesis in plants (Dastmalchi et al., 2018). We searched for CYP450 proteins in these three families from *S. acutum* and found 19 members. The 19 screened enzymes were subjected to phylogenetic analysis, together with their characterized homologs from *P. somniferum*, *C. japonica*, and *Berberis stolonifera* (**Figure 6A**). Seven unigenes encode proteins of the CYP80B subfamily, which catalyzes the hydroxylation of the 3′-position of (S)-*N*-methylcoclaurine. Two genes (gene40122 and gene27149) encode proteins of the CYP80G2 subfamily. Six genes (gene1888, gene24702, gene2351, gene2423, gene1693, and gene25895) encode the CYP82Y/N subfamily enzymes that are involved in the formation of 1,2-dehydroreticuline, protopine, dihydrosanguinarine. Gene46351 and gene30689 encode proteins of the CYP719A/B subfamilies. Salutaridine synthase (SalSyn) is a representative enzyme of the CYP719B1 family. It is responsible for the bridge-forming C–C phenol coupling of (*R*)-reticuline to yield the promorphinan alkaloid salutaridine. As sinomenine is also a member of the morphinan alkaloid with a different bridge from morphine, thebaine, and codeine, one of the enzymes encoded by gene46351 and gene30689 is proposed to catalyze the C–C phenol coupling of (*S*)-reticuline, instead of (*R*)-reticuline, to form sinomenine and its derivatives. The expression patterns of these genes were analyzed. It could be seen that genes encoding proteins of the CYP80B family were actively expressed in the root and leaf, while their expression levels were low in the stem (**Figure 6B**). The CYP719A/B family of proteins were expressed at a higher level in the root and stem than in the leaf and stem.

Plenty of *O*-methyltransferases (OMTs) and *N*-methyltransferases (NMTs) are involved in modifying the alkaloid scaffolds during the biosynthetic process of BIAs. We performed phylogenetic analysis and conserved motif structure prediction of the methyltransferases from *S. acutum* to mine enzymes catalyzing the methylation reactions in BIA biosynthesis (**Figure 6C**). The characterized methyltransferases from the BIA biosynthetic pathways were added to the phylogenetic tree for comparison. Three genes (gene41327, gene42607, and gene38655) probably encode 4′-OMTs that convert (*S*)-3-hydroxy-*N*-methylcoclaurine to (*S*)-reticuline. Gene41327 and gene38655 each encode a protein with a dimerization domain in the N-terminal. In contrast, no dimerization domain was detected in the enzyme encoded by gene42607. Gene expression analysis revealed that all these three genes are highly expressed in the leaf. Gene4475 encodes a 6-OMT that catalyzes the methylation of (*S*)-norcoclaurine to afford (*S*)-coclaurine. Six genes encode enzymes similar to CjCoOMT, a columbamine O-methyltransferase from *C. japonica*. Gene expression patterns showed that the expression levels of gene4552 and gene48928 were much higher in the root and stem than in the other two tissues. The scoulerine 9-*O*-methyltransferase (SOMT) converts (*S*)-scoulerine to (*S*)-tetrahydrocolumbamine in the formation of phthalideisoquinoline alkaloids. It can be seen from the phylogenetic analysis that three genes (gene 23249, gene36725, and gene42393) encode SOMTs in *S. acutum*. Gene36725 and gene42393 had similar expression patterns and were both actively expressed in the root and the leaf (**Figure 6D**). Gene23249 had the highest level in the leaf. Coclaurine *N*-methyltransferase (CNMT) catalyzes the methylation of (*S*)-coclaurine to generate (S)-N-methylcoclaurine. Three genes (gene35933, gene44271, and gene40181) encode proteins homologous to CNMTs from *P. somniferum* and *C. japonica*. Gene expression pattern analysis revealed that the root and stem of *S. acutum* had lower expression levels of these three genes, while the seed had the highest expression level (**Figure 6D**).

In order to check the reliability of the transcriptome and the DEG data, we selected four genes (gene4552, gene4645, gene29764, and gene30689) that encode enzymes of the CYP450 families or methyltransferases to compare their expression levels determined by qRT-PCR analysis and by the transcriptome data. For the qRT-PCR analysis, gene4552 had highest expression level in the stem and minimal levels in the other tissues. Gene4645, gene29764, and gene30689 all exhibited noticeable expression in two tissues (**Figure S5**). These results were consistent with the gene expression levels revealed in the transcriptome data (**Figures 6B**, **D**), showing that the gene expression levels determined by transcriptome sequencing were reliable.

**Figure 7:**
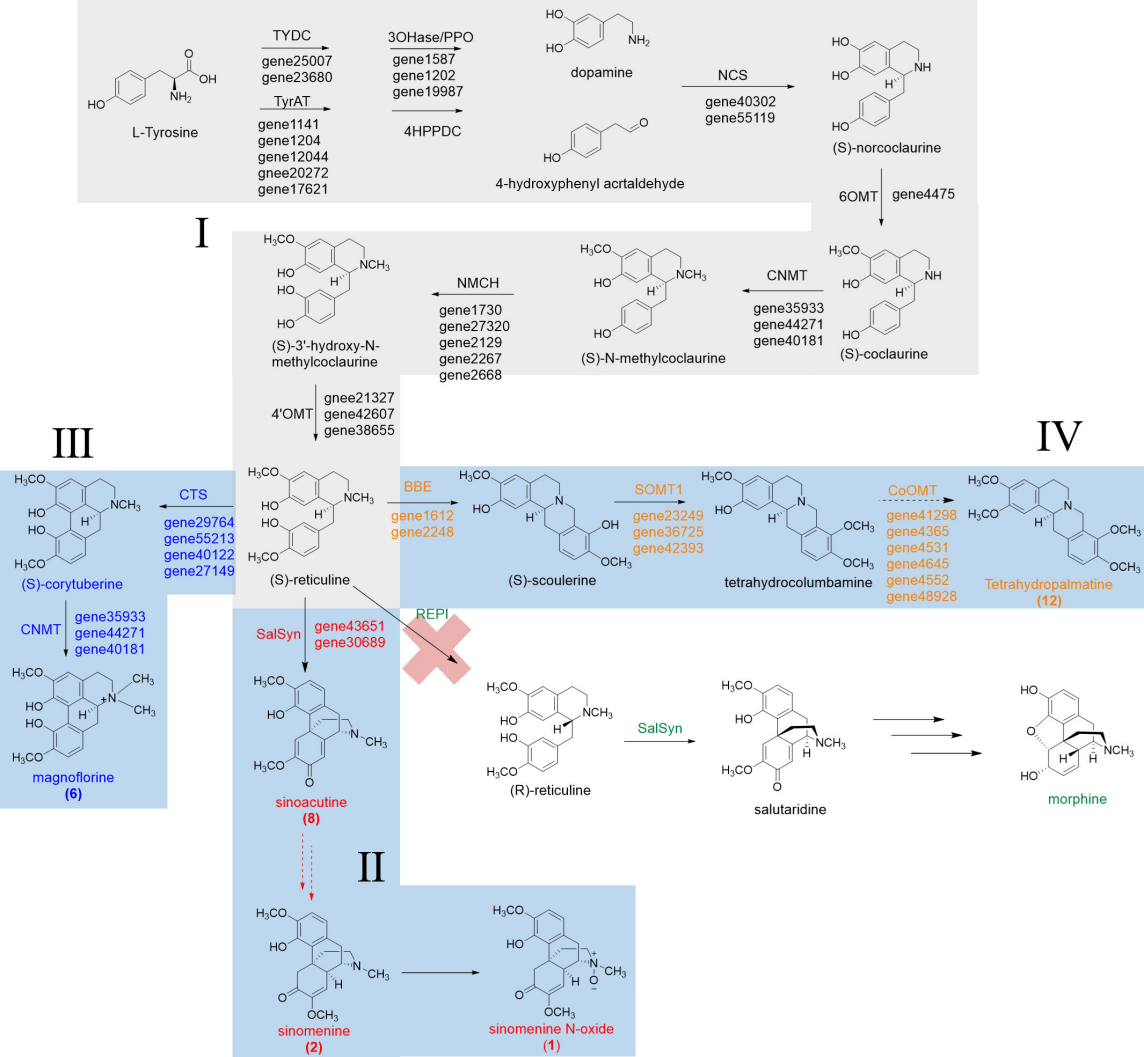
Proposed biosynthetic pathways for sinomenine, magnoflorine, and tetrahydropalmatine in *S. acutum*.

The epimerization of (*S*)-reticuline to (*R*)-reticuline is catalyzed by the reticuline epimerase (REPI, also known as STORR), and it is an important branch point in the biosynthesis of promorphinan/morphinan subclass of BIAs. Interestingly, we could not find orthologs of REPI from the transcriptome data of *S. acutum*, which is consistent with the lack of (*R*)-reticuline-derived alkaloids in this medicinal plant (**Figure 7**).

From the quantitative analysis of alkaloids, DEG- and function-based candidate gene screening, as well as the characterized biosynthetic pathways for BIAs, we were able to propose the biosynthetic pathways for a few BIAs in *S. acutum*, including the common biosynthetic pathway (route I), the pathway from (*S*)-reticuline to sinomenine and sinomenine *N*-oxide (route II), magnoflorine (route III), and tetrahydropalmatine (route IV) (**Figure 7**). Only a few candidate genes were proposed for each step of BIA biosynthesis in *S. acutum*, which should greatly facilitate the expression and function characterization of their encoded enzymes.

## 4 Discussion

### 4.1 A combination of SMRT and NGS generated a high-quality full-length transcriptome of *S. acutum*

BIAs are a large and diverse group of specialized plant secondary metabolites with important research and medicinal value (Weber and Opatz 2019). *Sinomenium acutum* is a with a long history of therapeutical applications. Sinomenine is a BIA from *S. acutum* and has been used clinically as an antirheumatic drug (Zhao et al., 2012). Recently, various pharmacological studies have shown the potential of sinomenine in treating cardiovascular diseases (Jiang et al., 2019; Yuan et al., 2021; Zhang et al., 2021). Besides sinomenine, more than 50 BIAs have been isolated from *S. acutum*. However, the biosynthetic pathways of BIAs from *S. acutum* remain elusive due to the lack of genomic or transcriptomic information.

With the development of high-throughput sequencing technology, transcriptome sequencing has become a powerful tool for studying the regulation of gene expression and biosynthetic pathways for specific metabolites (He et al., 2018; Xu et al., 2021). However, due to the limited read length of second-generation sequencing (SGS), the quality of transcripts obtained by this technology is often unsatisfactory. In contrast, the third-generation sequencing technology represented by PacBio utilizes SMRT sequencing technology to obtain high-quality, full-length transcripts, which efficiently improves the quality of the transcriptome data (He et al., 2018; McCombie et al., 2019). However, the high error rate of SMRT sequencing is not negligible. The combination of NGS and SMRT can generate full-length transcriptome data with high accuracy (Zhang et al., 2019). In this study, we constructed twelve high-quality RNA libraries of *S. acutum*, including the root, stem, leaf, and seed tissues. The full-length transcriptome data was obtained by combining the SMRT and NGS sequencing techniques. In the absence of a reference genome of *S. acutum*, the high-quality full-length transcriptome data can significantly facilitate the characterization of the secondary metabolite biosynthetic pathways in *S. acutum*.

With the transcriptome data in hand, we annotated the functions of the unigenes using the NR, GO, KOG, and KEGG databases. More than 86% of the unigenes could be annotated in at least one public database, and around one-third of the unigenes were co-annotated in all five databases. In the NR database, 25.88% and 21.25% of the annotated unigenes matched the sequences of *M. cordata* and *N. nucifera*, respectively. It is well-known that *M. cordata* and *N. nucifera* both accumulate plenty of BIAs. Detailed characterization of the matched unigenes among these three species would promote our understanding of the regulation and biosynthesis of BIAs in plants.

### 4.2 The DEG and WGCNA analysis facilitate the screening of candidate genes for BIA biosynthesis

Although the function annotation using public protein databases can assign the possible function of unigenes from the transcriptome data, screening of the candidate genes involved in BIA biosynthesis still needs more information to differentiate the actual enzymes from their respective homologs. WGCNA is now widely used in the mining of genomic data. The WGCNA algorithm assumes that the gene network follows the scale-free topology rule and generates the gene co-expression matrix. Unlike the traditional gene-to-phenotype mode, which relates individual genes to phenotype, WGCNA focuses on the relationship between a few simplified modules and the traits. The WGCNA analysis can efficiently alleviate multiple inherent problems associated with microarray data analysis (Fuller et al., 2007). The genes in the same module possess a high co-expression similarity, while genes in different modules have a low co-expression similarity.

To reduce the scale of the network, we selected seven representative alkaloids from *S. acutum*, i.e., menisperine, sinomenine, tetrahydropalmatine, sinomenine *N*-oxide, sinoacutine, magnoflorine, and acutumine, to correlate these metabolites with specific modules. In this study, seven modules were detected based on the co-expression network. We found that the pink, purple, and dark red modules correlate much more with the accumulation of the seven BIAs than the other four. We also observed that the pink module had obvious adjacency with the purple and dark red modules, while the purple and dark red modules showed no adjacency to each other. It is reasonable to propose that the unigenes in the pink, purple, and dark red module have a higher possibility of participating in the biosynthesis of BIAs in *S. acutum*.

Analysis of the expression patterns of genes in different tissues can further help to reduce the number of candidate genes. Gene expression profiles were compared among the root, stem, leaf, and seed tissues. Since the root of *S. acutum* has the highest alkaloid contents and the leaf has the lowest contents, we compared the gene expression patterns in these two tissues and observed 8,270 DEGs. Further analysis of unigenes enriched in KEGG pathway terms revealed a total of 47 unigenes that mapped to the “isoquinoline biosynthetic pathway” category (ko00950). Proteins encoded by these 47 genes were subjected to more detailed function annotation and expression analysis. It could be concluded from the process that combining DEG and WGCNA analysis is an efficient approach to mining the potential genes involved in specific secondary metabolites.

### 4.3 Proposal of the BIA biosynthetic pathways in *S. acutum*

BIAs are mainly isolated from plants of the Ranunculales order, such as the Papaveraceae, Ranunculaceae, Berberidaceae, and Menispermaceae families (Hagel and Facchini, 2013). The common biosynthetic pathway for isoquinoline alkaloids has been well-studied in *P. somniferum*, *C. japonica*, *N. nucifera*, and *M. cordata* (Beaudoin and Facchini, 2014). Production of many characterized BIAs follows the common route, which starts from the condensation of dopamine and 4-hydroxyphenylacetaldehyde to (*S*)-norcoclaurine and ends at the formation of (*S*)-reticuline. (*S*)-reticuline is a key branch point intermediate to the generation of BIAs. It can undergo different reactions, such as aromatic ring hydroxylation, C–C or C–O coupling, and *O*- or *N*-methylation, to yield the vast structural diversity of BIAs. In this study, we screened 24 genes that encode proteins participating in the biosynthetic route from dopamine and 4-hydroxyphenylacetaldehyde to (*S*)-reticuline. Enzymes of the CYP450 family and methyltransferase family are the key players in converting (*S*)-reticuline to different BIAs. Candidate proteins involved in these steps were mined using the Pfam annotation and phylogenetic analysis using the characterized orthologs as references, including the CYP80, CYP82, and CYP719 subfamilies, as well as orthologs from the 4′-OMT, 6-OMT, CoOMT, SOMT, and CNMT subclasses. With the collected candidate enzymes in hand, we could propose the biosynthetic pathways for some of the BIAs, i.e., sinomenine, sinoacutine, magnoflorine, and tetrahydropalmatine.

Two points are worth mentioning from this study: a) although the root has much higher concentrations for most of the characterized BIAs herein, plenty of the screened genes were not expressed at the highest levels in the root. This phenomenon could be attributed to the spatial and temporal-specific production of some BIAs or their biosynthetic precursors in tissues other than the root. After the biosynthesis, the final or intermediate BIAs could be transported from the different tissues to the root. b) REPI can convert (*S*)-reticuline to (*R*)-reticuline, which is an essential step in the biosynthesis of some pharmaceutically important morphinan BIAs, such as morphine and codeine. However, this enzyme was not annotated in the transcriptome of *S. acutum*. Therefore, conversion of the (*S*)-reticuline intermediate to (*R*)-reticuline does not occur in this plant. This result is consistent with the observation that no (*R*)-reticuline-derived BIAs have been isolated from *S. acutum*.

## 5 Conclusions

*Sinomenium acutum* is an important medicinal plant with a long history of application. It can produce more than 50 BIAs. However, the biosynthetic pathways of BIAs in *S. acutum* remain elusive. In the current study, we analyzed the contents of 12 BIAs in four different tissues of *S. acutum*. We obtained its high-quality full-length transcriptome data by combining the NGS and SMRT sequencing. Annotation of the transcripts resulted in 60,675 unigenes. The candidate genes responsible for BIA biosynthesis were mined by the WGCNA and DEG analysis and the KEGG pathway enrichment analysis. Based on the functions of the screened candidate genes, we were able to propose the biosynthetic pathways for some of the BIAs in *S. acutum*, including sinomenine, sinoacutine, magnoflorine, and tetrahydropalmatine. Our work lays the foundation for the characterization of the enzymes involved in BIA biosynthesis and will benefit the heterologous production of high-value BIAs in microbial cell factories.

## Data availability statement

The original contributions presented in the study are publicly available. This data can be found here: NCBI, PRJNA843226.

## Author contributions

YY: Methodology, data analysis, and writing. YS: Material collection, data analysis, and writing. ZW: Data analysis and writing. MY: Methodology and data analysis. RS: Material collection and data analysis. LX: Material collection. XH: Material collection and conceptualization. CW: Conceptualization and data analysis. XY: Conceptualization, funding acquisition, data analysis, project administration, and writing-reviewing. All authors contributed to the article and approved the submitted version.
