# Supplementary material for: Full-length transcriptome and metabolite analysis reveal reticuline epimerase-independent pathways for benzylisoquinoline alkaloids biosynthesis in Sinomenium acutum
Source: Front Plant Sci. 2022 Dec 20;13:1086335. doi: 10.3389/fpls.2022.1086335 (PMC9808091; doi:10.3389/fpls.2022.1086335)
Supplement: Supplementary file 1 [file DataSheet_1.docx]

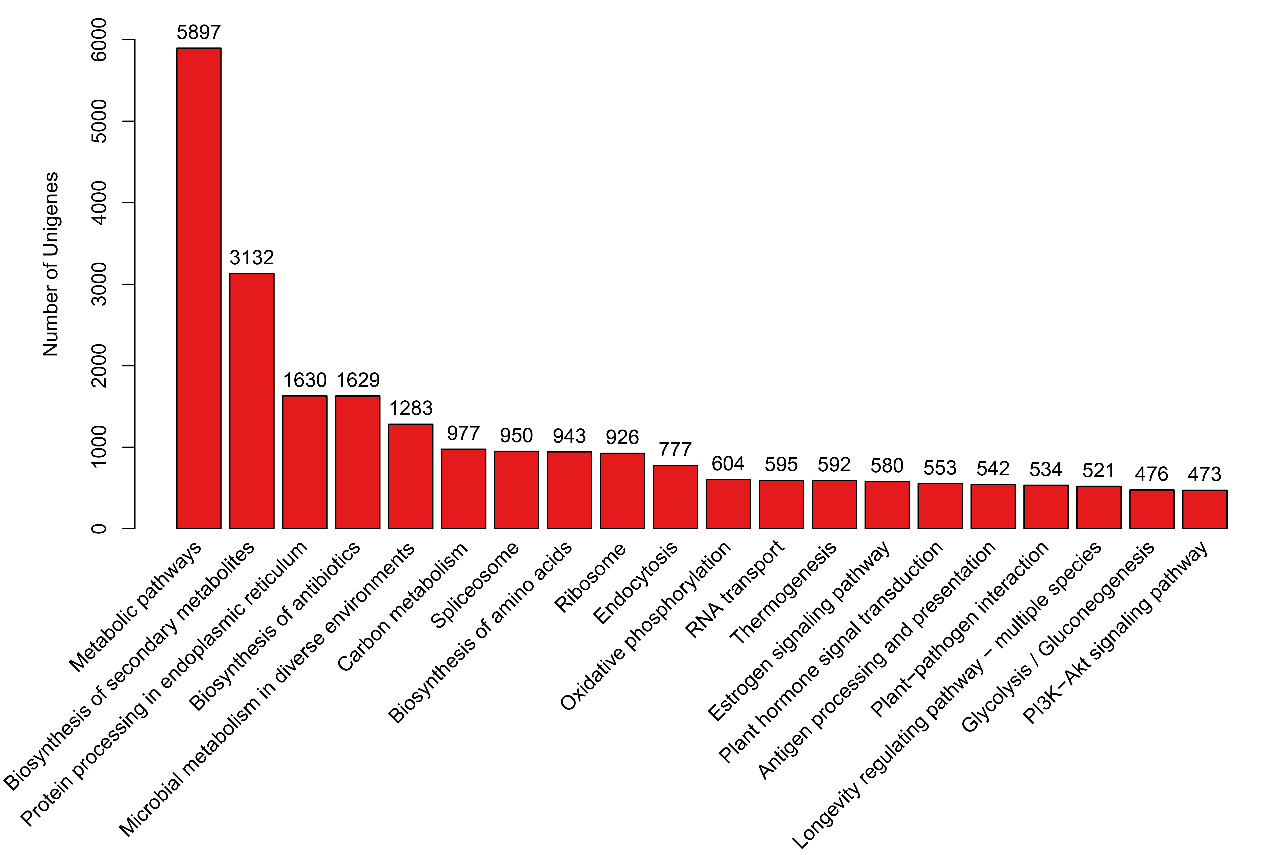


**Figure S1.** Bar graph of the top 20 KEGG annotation pathways in *S. acutum*

**Figure S2.** GO annotation of the unigenes in *S. acutum*


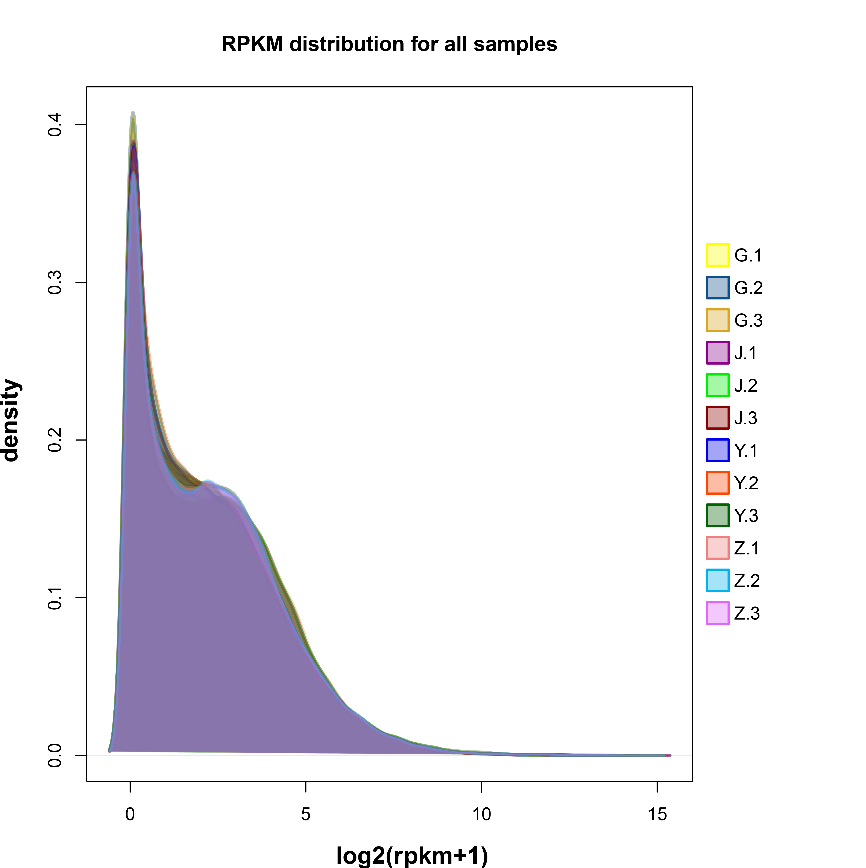


**Figure S3.** The RPKM distribution of different tissue samples


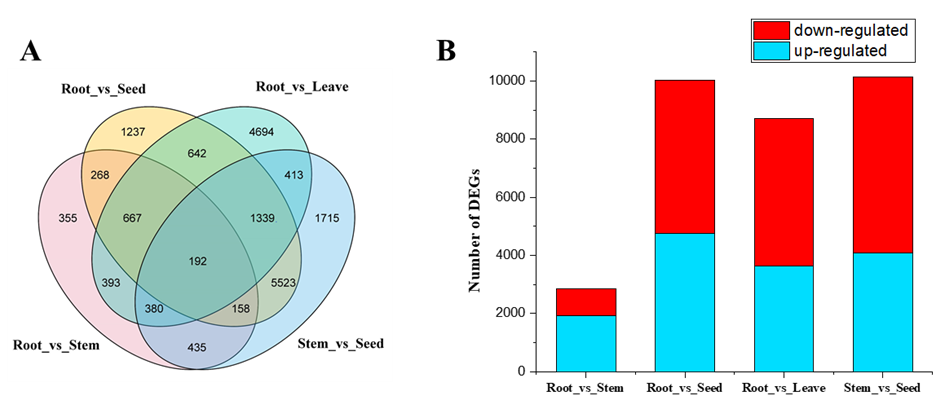


**Figure S4** DEGs gene expression in root, stem, and seed tissues of *S. acutum* （A）Venn diagram of differentially expressed genes in root, stem, leaf, and seed tissues（B）number of differential genes in the root, stem, leaf and seed tissues


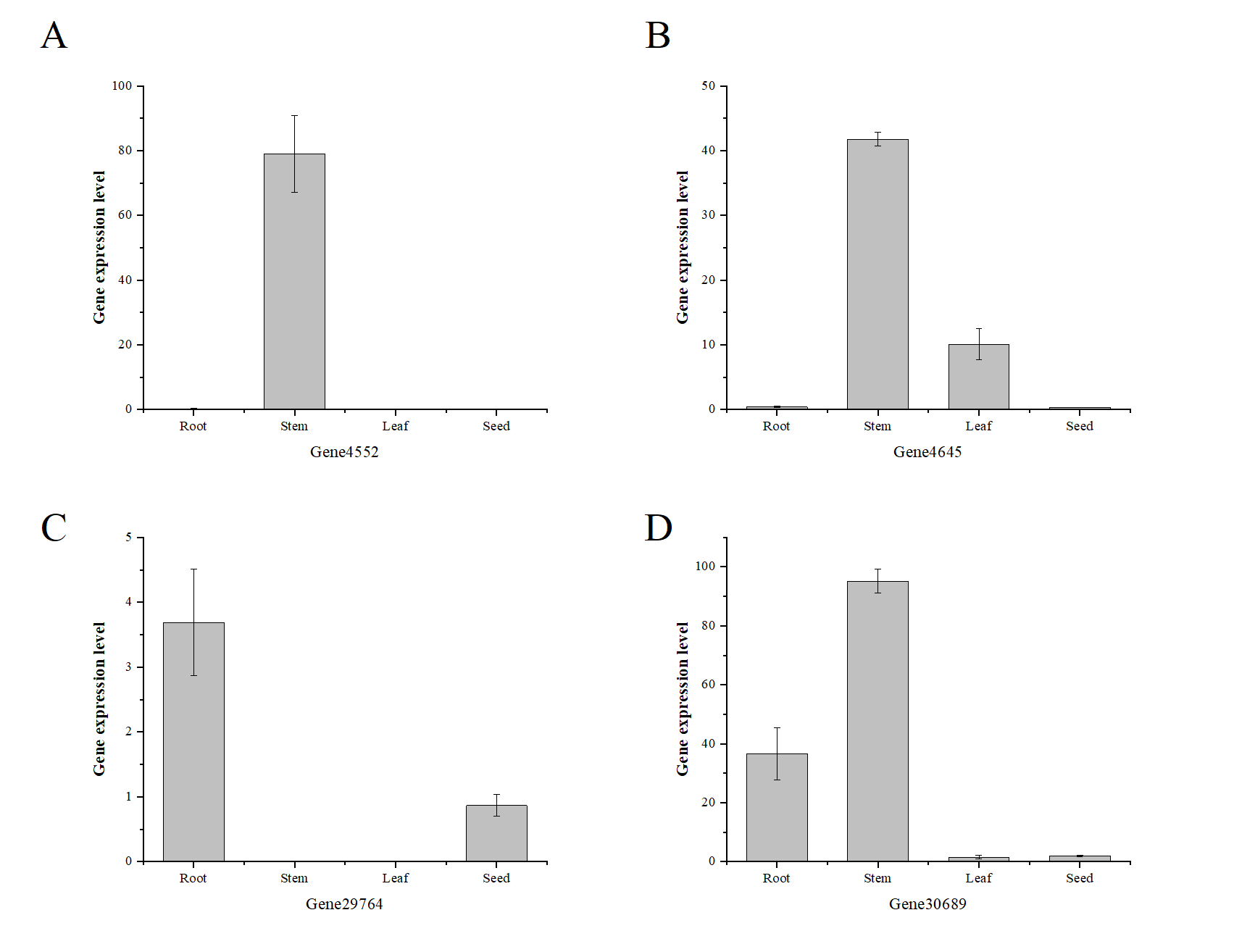


**Figure S5** qRT-PCR analysis of the expression levels of four selected genes involved in BIAs biosynthesis.

| **Table S1. The secondary metabolite pathway in *S. acutum*** | |
| --- | --- |
| Secondary Metabolites Pathway | Unigene number |
| Phenylpropanoid biosynthesis | 247 |
| Tropane, piperidine and pyridine alkaloid biosynthesis | 61 |
| Flavonoid biosynthesis | 59 |
| Isoquinoline alkaloid biosynthesis | 47 |
| Streptomycin biosynthesis | 47 |
| Monobactam biosynthesis | 29 |
| Prodigiosin biosynthesis | 26 |
| Glucosinolate biosynthesis | 19 |
| Stilbenoid, diarylheptanoid and gingerol biosynthesis | 17 |
| Neomycin, kanamycin and gentamicin biosynthesis | 15 |
| Phenazine biosynthesis | 13 |
| Betalain biosynthesis | 12 |
| Novobiocin biosynthesis | 9 |
| Aflatoxin biosynthesis | 8 |
| Caffeine metabolism | 7 |
| Anthocyanin biosynthesis | 7 |
| Flavone and flavonol biosynthesis | 7 |
| Acarbose and validamycin biosynthesis | 1 |
| Isoflavonoid biosynthesis | 1 |

**Table S2. Information about the primer sequences, the annealing temperature of the primers, and the expected sizes of the amplified products in the qRT-PCR analysis.**

| gene | Primer sequence | Tm (°C) | Expected  size (bp) |
| --- | --- | --- | --- |
| β-actin | CCTCTTCCAGCCTTCCTTCAT | 55 | 100 |
|  | TCTCCTTGCTCATCCTGCAG |  |  |
| Gene4552 | AGGGGTTGATGAGGGAGAATGT | 60 | 210 |
|  | CCGCACGGGGAATAGAATG |  |  |
| Gene4645 | CTATGAGAAGATGGAGAAGGA | 55 | 297 |
|  | TTGGGAATAGAATGGAACA |  |  |
| Gene29764 | ATAGCCGCTTTCTTTCCCATTTTC | 65 | 269 |
|  | AGCTCCGACATTACCCACTCCAC |  |  |
| Gene30689 | CTACTCTCGCAGGGGTTCAG | 65 | 265 |
|  | ACGGTGCTATCGGTTTCATC |  |  |
